# COVID-19 waves in an urban setting 2020–2022: an electronic medical record analysis

**DOI:** 10.3389/fpubh.2024.1323481

**Published:** 2024-01-29

**Authors:** Yi-shuan Elaine Chen, Susan H. Gawel, Pankaja Desai, Juan Rojas, Hannah J. Barbian, Nagarjuna Tippireddy, Rajkamal Gopinath, Sharon Schneider, Anthony Orzechowski, Gavin Cloherty, Alan Landay

**Affiliations:** ^1^Abbott Diagnostics Division, Abbott Laboratories, Abbott Park, IL, United States; ^2^Abbott Pandemic Defense Coalition, Abbott Park, IL, United States; ^3^Rush University Medical Center, Chicago, IL, United States

**Keywords:** SARS-CoV-2, COVID-19, mortality, electronic health records, surveillance

## Abstract

**Background:**

Global and national surveillance efforts have tracked COVID-19 incidence and clinical outcomes, but few studies have compared comorbid conditions and clinical outcomes across each wave of the pandemic. We analyzed data from the COVID-19 registry of a large urban healthcare system to determine the associations between presenting comorbidities and clinical outcomes during the pandemic.

**Methods:**

We analyzed registry data for all inpatients and outpatients with COVID-19 from March 2020 through September 2022 (*N* = 44,499). Clinical outcomes were death, hospitalization, and intensive care unit (ICU) admission. Demographic and clinical outcomes data were analyzed overall and for each wave. Unadjusted and multivariable logistic regressions were performed to explore the associations between age, sex, race, ethnicity, comorbidities, and mortality.

**Results:**

Waves 2 and 3 (Alpha and Delta variants) were associated with greater hospitalizations, ICU admissions, and mortality than other variants. Chronic pulmonary disease was the most common comorbid condition across all age groups and waves. Mortality rates were higher in older patients but decreased across all age groups in later waves. In every wave, mortality was associated with renal disease, congestive heart failure, cerebrovascular disease, diabetes, and chronic pulmonary disease. Multivariable analysis found that liver disease and renal disease were significantly associated with mortality, hospitalization, and ICU admission, and diabetes was significantly associated with hospitalization and ICU admission.

**Conclusion:**

The COVID-19 registry is a valuable resource to identify risk factors for clinical outcomes. Our findings may inform risk stratification and care planning for patients with COVID-19 based on age and comorbid conditions.

## 1 Introduction

The COVID-19 pandemic continues, with more than 750 million individuals infected and more than 7 million deaths reported worldwide as of June 22, 2023 (1). The pandemic has been marked by waves of infection, driven by the evolution of the SARS-CoV-2 virus over time. Global and national surveillance efforts have documented the incidence of COVID-19 and its clinical outcomes, as well as the changes in dominant circulating SARS-CoV-2 variants in each wave (2). Throughout the pandemic, several studies have compared outcomes between early or late phases or between variant waves, but few studies have examined outcomes longitudinally across waves of SARS-Cov-2 variants. Four large studies have reported differences in inpatient outcomes (3), perinatal outcomes (4), ECMO-related outcomes (5), and outcomes in children diagnosed with multisystem inflammatory syndrome (MIS-C) (6) across multiple SARS-CoV-2 waves. A review by Lin et al. (7) found wild-type SARS-CoV-2 infection has less severe outcomes (e.g., intensive care unit [ICU] admission, hospitalization, and death) compared to other variants including Alpha, Delta, Gamma, and Beta. Several studies have found Omicron infection to have less severe outcomes including hospital admissions, hospital admissions for symptoms, ICU admission, ventilation, and death, compared to Delta (8–14). Studies are needed analyze outcomes by variant and risk factors. The primary objective of this study was to determine the associations between comorbidities in the Charlson Comorbidity Index (15) and mortality among patients with COVID-19 seen in an urban healthcare system in the United States, across all waves and for each wave of the pandemic from March 2020 to September 2022. As a secondary objective, we aimed to evaluate these same factors and their association with hospital admission and ICU admission.

## 2 Methods

### 2.1 Study participants and registry development

In partnership with Abbott Pandemic Defense Coalition (APDC), Rush University Medical Center (RUMC, Chicago, IL) established a COVID-19 registry to collect RUMC electronic health record (EHR) data on infections, patient demographics, and clinical outcomes. The Abbott Pandemic Defense Coalition (APDC) is a global multisector scientific and public health partnership whose primary objective is the early detection and mitigation of infectious disease threats of pandemic potential (16).

Each patient in the registry is assigned an identification number to protect privacy and deidentified data from the COVID-19 registry was analyzed for this study. The COVID-19 registry was developed utilizing Epic electronic health records data at RUMC. Specific data elements were first determined to develop the structure of the registry. SQL was used to conduct data extraction and organization of the data. The data was mapped to the Observational Medical Outcomes Partnership Common Data Model (OMOP) Common Data Model^1^ release 5.3.1 (17). The registry includes variables such as demographic characteristics, conditions, and COVID-19 testing information and related outcomes. This study was approved by the Institutional Review Board of Rush University Medical Center.

We analyzed data in the registry for all inpatients and outpatients who were under investigation for COVID-19 from March 2020 through September 2022 (*N* = 44,499). Patients were considered under investigation if they were tested for COVID-19 when they presented for care at RUMC. COVID-19 positivity was defined as any documented infection (“Infection of upper respiratory tract caused by 2019 novel coronavirus”: OMOP condition concept ID 37310286) (17). COVID-19 positivity was defined as any documented infection (“Infection of upper respiratory tract caused by 2019 novel coronavirus”: OMOP condition concept ID 37310286) (17). History of comorbidities using the Charlson Comorbidity Index (CCI) were identified if the patient ever had a corresponding International Classification of Diseases (ICD) code for the condition. The clinical outcomes of death, hospitalization, and intensive care unit (ICU) admission were recorded. Inpatient deaths were defined as deaths associated with an inpatient or emergency room visit. Outpatient deaths were defined as deaths associated with an outpatient or patient vehicle encounter, with the date of death identified through the patient EHR. Other demographic and clinical information were also captured for each patient.

### 2.2 Wave definitions

Demographic and clinical outcomes data were analyzed overall and for each wave of the COVID-19 pandemic. Each wave was characterized by the predominant circulating SARS-CoV-2 variant in the Chicago area, based on surveillance sequencing data from the Abbott Global Viral Surveillance Program, as follows: Wave 1, March 7, 2020 to March 20, 2021, Wildtype+D614G; Wave 2: March 21, 2021 to June 19, 2021, Alpha variant; Wave 3: June 20, 2021 to December 11, 2021, Delta variant; Wave 4: December 12, 2021 to March 19, 2022, Omicron BA.1 variant; Wave 5: March 20, 2022 to June 18, 2022, Omicron BA.2 variant; and Wave 6: June 19, 2022 to present (September 30, 2022), Omicron BA.4/BA.5 variant.

### 2.3 Statistical analysis

Descriptive statistics were used to summarize COVID-19 registry data on patient demographics (age, sex, race, and ethnicity), comorbidities, hospitalization, ICU admissions, and deaths across all waves (overall) and by wave. Categorical variables were represented by proportion and continuous variables by medians and interquartile ranges (IQRs). Unadjusted and forward stepwise multivariable logistic regressions were performed to explore the associations between demographic variables (age, sex, race, ethnicity), comorbidities (as defined by the Charlson Comorbidity Index: congestive heart failure, peripheral vascular disease, cerebrovascular disease, dementia, chronic pulmonary disease, rheumatic disease, peptic ulcer disease, mild liver disease, diabetes without chronic complications, diabetes with chronic complications, hemiplegia or paraplegia, renal disease, cancer (any malignancy), moderate/severe liver disease, metastatic solid tumor, AIDS/HIV), and the three outcomes of hospitalization, ICU admissions, and deaths. Models reported odds ratios (OR) and 95% confidence intervals (CI) and a *p* < 0.05 was used to identify statistically significant variables for forward selection. Age-stratified analyses were conducted to identify differences in demographics and comorbidities for all COVID-19 patients and those reported as a death across age groups (18–49 years, 50–69 years, and 70–90 years) overall and by wave. No imputations were made for missing data and all analyses were conducted using R4.1.2.

## 3 Results

### 3.1 Demographics of registry patients across all waves of the pandemic

A total of 44,499 patients who tested positive for COVID-19 from March 2020 to September 2022 were included in this registry analysis (Table 1). More than 75% (*n* = 34,530) of the population was 18–69 years of age. Patients were majority female (*n* = 25,089, 56.4%), with a similar proportion of White (*n* = 15,028, 33.8%) and Black or African American (*n* = 13,258, 29.8%) patients. Approximately one-third (n = 14,866, 33.4%) of patients in the registry did not report a racial group. The top 5 prevalent comorbidities in the study population were chronic pulmonary disease (*n* = 5,057, 11.4%), diabetes without chronic complications (*n* = 3,529, 7.9%), renal disease (*n* = 2,263, 5.1%), congestive heart failure (*n* = 2,123, 4.8%), and diabetes with chronic complications (*n* = 2089, 4.7%).

**Table 1:** Study population demographics (*N* = 44,499).

	n	%
Age (years)		
<18	5,848	13.1
18–49	23,990	53.9
50–69	10,540	23.7
70–90	3,860	8.7
≥90	261	0.6
Sex		
Female	25,089	56.4
Male	19,410	43.6
Race		
White	15,028	33.8
Black or African American	13,258	29.8
Asian	1,157	2.6
American Indian or Alaska Native	113	0.3
Native Hawaiian or Other Pacific Islander	77	0.2
Undisclosed	14,866	33.4
Comorbidity		
Chronic pulmonary disease	5,057	11.4
Diabetes without chronic complications	3,529	7.9
Renal disease	2,263	5.1
Congestive heart failure	2,123	4.8
Diabetes with chronic complications	2089	4.7
Cerebrovascular disease	1849	4.2
Cancer (any malignancy)	1842	4.1
Peripheral vascular disease	1,599	3.6
Mild liver disease	1,453	3.3
Myocardial infarction	1,092	2.5
Rheumatic disease	609	1.4
Dementia	560	1.3
Metastatic solid tumor	509	1.1
Peptic ulcer disease	251	0.6
Hemiplegia or paraplegia	218	0.5
Moderate/severe liver disease	200	0.5
AIDS/HIV	143	0.3

### 3.2 Demographics of patients with hospitalization, ICU admission, and death across all waves

#### 3.2.1 Demographics for overall hospitalization

Of the 44,499 COVID-19–positive patients in the registry, 13,454 (30.2%) were reported as hospitalized across all waves of the pandemic (Table 2). Of patients who were hospitalized, 44.9% (*n* = 6,042) were 18–49 years of age, 52.2% (*n* = 7,029) were female, 27.3% (*n* = 3,673) were White, 42.1% (*n* = 5,659) were Black or African American, 14.3% (*n* = 1922) had chronic pulmonary disease, and 11.4% (*n* = 1,539) had diabetes without chronic complications (Table 3). The highest hospitalization rate was seen in the oldest age group (>90 years; *n* = 162, 62.1%), followed by 70–90 years (*n* = 1937, 50.2%) and 50–69 years (*n* = 3,376, 32.0%). The hospitalization rate was higher in males than females (33.1% vs. 28.0%) and was highest for Black or African American patients (42.7%). The next highest hospitalization rates were for American Indian or Alaska Natives (*n* = 29, 25.7%), followed by White patients (24.4%). The top 5 most common comorbidities in hospitalized patients were chronic pulmonary disease, diabetes without chronic complications, renal disease, congestive heart failure, and diabetes with chronic complications (Table 3).

**Table 2:** Characteristics overall and by outcome (*N* = 44,499).

Characteristic	N (%)	Hospitalized n (%)	ICU n (%)	Death n (%)
Age (years)				
<18	5,848 (13.1%)	1937 (33.1%)	72 (1.2%)	0 (0%)
18–49	23,990 (53.9%)	6,042 (25.2%)	469 (2.0%)	125 (0.5%)
50–69	10,540 (23.7%)	3,376 (32.0%)	798 (7.6%)	404 (3.8%)
70–90	3,860 (8.7%)	1937 (50.2%)	519 (13.4%)	453 (11.7%)
>90	261 (0.6%)	162 (62.1%)	32 (12.3%)	64 (24.5%)
Gender				
Female	25,089 (56.4%)	7,029 (28.0%)	780 (3.1%)	475 (1.9%)
Male	19,410 (43.6%)	6,425 (33.1%)	1,110 (5.7%)	571 (2.9%)
Race				
White	15,028 (33.8%)	3,673 (24.4%)	573 (3.8%)	415 (2.8%)
Black or African American	13,258 (29.8%)	5,659 (42.7%)	676 (5.1%)	316 (2.4%)
Asian	1,157 (2.6%)	236 (20.4%)	46 (4.0%)	26 (2.2%)
American Indian or Alaska Native	113 (0.3%)	29 (25.7%)	3 (2.7%)	4 (3.5%)
Native Hawaiian or Other Pacific Islander	77 (0.2%)	16 (20.8%)	4 (5.2%)	2 (2.6%)
Undisclosed	14,866 (33.4%)	3,841 (25.8%)	588 (4.0%)	283 (1.9%)
Overall	44,499	13,454 (30.2%)	1890 (4.2%)	1,046 (2.4%)

**Table 3:** Frequency of comorbid conditions among patients with hospitalization, ICU admission, or death (*N* = 44,499).

Comorbidity	N (%)	Hospitalized n (%)	ICU n (%)	Death n (%)
Myocardial infarction	1,092 (2.5%)	647 (59.2%)	255 (23.4%)	165 (15.1%)
Congestive heart failure	2,123 (4.8%)	1,267 (59.7%)	427 (20.1%)	288 (13.6%)
Peripheral vascular disease	1,599 (3.6%)	863 (54.0%)	254 (15.9%)	187 (11.7%)
Cerebrovascular disease	1849 (4.2%)	1,025 (55.4%)	361 (19.5%)	255 (13.8%)
Dementia	560 (1.3%)	394 (70.4%)	80 (14.3%)	99 (17.7%)
Chronic pulmonary disease	5,057 (11.4%)	1922 (38.0%)	395 (7.8%)	228 (4.5%)
Rheumatic disease	609 (1.4%)	243 (39.9%)	57 (9.4%)	38 (6.2%)
Peptic ulcer disease	251 (0.6%)	132 (52.6%)	38 (15.1%)	32 (12.7%)
Mild liver disease	1,453 (3.3%)	532 (36.6%)	147 (10.1%)	99 (6.8%)
Diabetes without chronic complication	3,529 (7.9%)	1,539 (43.6%)	432 (12.2%)	208 (5.9%)
Diabetes with chronic complication	2089 (4.7%)	1,140 (54.6%)	366 (17.5%)	239 (11.4%)
Hemiplegia or paraplegia	218 (0.5%)	135 (61.9%)	55 (25.2%)	23 (10.6%)
Renal disease	2,263 (5.1%)	1,379 (60.9%)	430 (19.0%)	319 (14.1%)
Malignancy (including lymphoma and leukemia, except malignant neoplasm of skin)	1842 (4.1%)	660 (35.8%)	180 (9.8%)	152 (8.3%)
Moderate or severe liver disease	200 (0.5%)	134 (67%)	48 (24.0%)	45 (22.5%)
Metastatic solid tumor	509 (1.1%)	197 (38.7%)	59 (11.6%)	115 (22.6%)
AIDS/HIV	143 (0.3%)	64 (44.8%)	11 (7.7%)	7 (4.9%)
Overall	44,499	13,454 (30.2%)	1890 (4.2%)	1,046 (2.4%)

#### 3.2.2 Demographics for overall ICU admissions

Of the 44,499 COVID-19–positive patients in the registry, 1890 (4.2%) were reported as ICU admissions across all waves of the pandemic (Table 2). Of patients who were admitted to the ICU, 42.2% (*n* = 798) were 50–69 years of age, 58.7% (*n* = 1,110) were male, 30.3% (*n* = 573) were White, 35.8% (*n* = 676) were Black or African American, 22.9% (*n* = 432) had diabetes without chronic complications, and 22.8% (n = 430) had renal disease (Table 3). The highest ICU admission rate was seen in the 70–90 age group (*n* = 519, 13.4%) followed by the older than 90 years age group (*n* = 32, 12.3%) and 50–69 years (*n* = 798, 7.6%). The ICU admission rate was higher in males than females (5.7% vs. 3.1%) and was highest for Native Hawaiian and Other Pacific Islander (*n* = 77, 5.2). The next highest ICU admission rates were for Black or African American patients (5.1%), followed by Asian patients (*n* = 46, 4.0%). The top 5 most common comorbidities in patients admitted to the ICU were diabetes without chronic complications, renal disease, congestive heart failure, chronic pulmonary disease, and diabetes with chronic complications (Table 3).

#### 3.2.3 Demographics for overall mortality

Of the 44,499 COVID-19–positive patients in the registry, 1,046 (2.4%) were reported as a death across all waves of the pandemic (Table 2). Deaths were reported for 855 (81.7%) inpatients and 191 (18.3%) outpatients. Of patients who died, 43.3% (*n* = 453) were 70–90 years of age, 54.6% (*n* = 571) were male, 39.7% (*n* = 415) were White, 30.5% (*n* = 319) had renal disease, and 27.5% (*n* = 288) had congestive heart failure. The majority (*n* = 620; 59.3%) of decedents had 2 or more comorbidities reported; 223 (21.3%) had 1 comorbidity and 203 (19.4%) had no comorbidities. The highest mortality rate was seen in the oldest age group (>90 years; *n* = 64, 24.5%), followed by 70–90 years (*n* = 453, 11.7%), and 50–69 years (*n* = 404; 3.8%). The mortality rate was higher in males than females (2.9% vs. 1.9%) and was highest for American Indian or Alaska Natives (3.5%), though the sample size was small (*n* = 4/113). The next highest mortality rates were for White patients (2.8%), followed by Native Hawaiian or Other Pacific Islander (*n* = 2, 2.6%). The top 5 most common comorbidities in patients reported as a death were renal disease, congestive heart failure, cerebrovascular disease, diabetes with chronic complications, and chronic pulmonary disease (Table 3).

### 3.3 Hospitalization, ICU admission, and death in each wave of the pandemic

#### 3.3.1 Hospitalizations by wave

**Figure 1:**
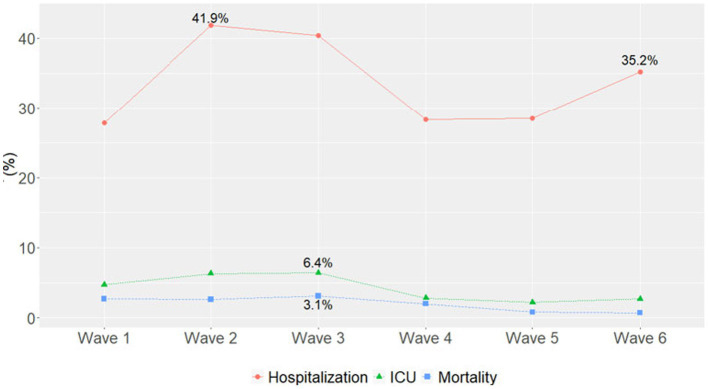
Percentage of hospitalizations, ICU admissions, and mortality in each pandemic wave. Wave 1: March 7, 2020 to March 20, 2021, Wildtype+D614G; Wave 2: March 21, 2021 to June 19, 2021, Alpha variant; Wave 3: June 20, 2021 to December 11, 2021, Delta variant; Wave 4: December 12, 2021 to March 19, 2022, Omicron BA.1 variant; Wave 5: March 20, 2022 to June 18, 2022, Omicron BA.2 variant; and Wave 6: June 19, 2022 to present (September 30, 2022), Omicron BA.4/BA.5 variant.

The highest number of hospitalizations occurred in Wave 1 (*n* = 6,365, 47.3%), followed by Wave 4 (Omicron BA.1 variant; *n* = 2,874, 21.4%) (Table 4). The hospitalization rate ranged from 41.9% in Wave 2 to 27.8% in Wave 1, with similar rates in Waves 4 and 5 (28.3 and 28.5%, respectively). The highest percentage of hospitalizations occurred in Wave 2, with the Alpha variant (*n* = 928, 41.9%) (Figure 1).

**Table 4:** Outcome by wave.

Wave: predominant SARS-CoV-2 variant (date range)	N (%)	Hospitalized n (%)	ICU n (%)	Death n (%)
1: Wildtype+D614G (3/7/2020–3/20/2021)	22,920 (51.5%)	6,365 (27.8%)	1,086 (4.7%)	627 (2.7%)
2: Alpha (3/21/21–6/19/2021)	2,216 (5.0%)	928 (41.9%)	140 (6.3%)	57 (2.6%)
3: Delta (6/20/2021–12/11/2021)	3,750 (8.4%)	1,515 (40.4%)	241 (6.4%)	118 (3.1%)
4: Omicron BA.1 (12/12/2021–3/19/2022)	10,155 (22.8%)	2,874 (28.3%)	288 (2.8%)	204 (2.0%)
5: Omicron BA.2 (3/20/2022–6/18/2022)	2,229 (5.0%)	636 (28.5%)	49 (2.2%)	18 (0.8%)
6: Omicron BA.4/BA.5 (6/19/2022–9/30/2022)	3,229 (7.3%)	1,136 (35.2%)	86 (2.7%)	22 (0.7%)
Overall	44,499	13,454 (30.2%)	1890 (4.2%)	1,046 (2.4%)

#### 3.3.2 ICU admissions by wave

Most ICU admissions occurred in Wave 1 (*n* = 1,086, 57.5%), followed by Wave 4 (Omicron BA.1 variant; *n* = 288, 15.2%) (Table 4). The ICU admission rate ranged from 6.4% in Wave 3 to 2.2% in Wave 5. The highest percentage of ICU admissions occurred in Wave 3 with the Delta variant (*n* = 241, 6.4%) (Figure 1). ICU admission rates continued to decline during Omicron waves and maintained similar trajectories.

#### 3.3.3 Mortality by wave

**Figure 2:**
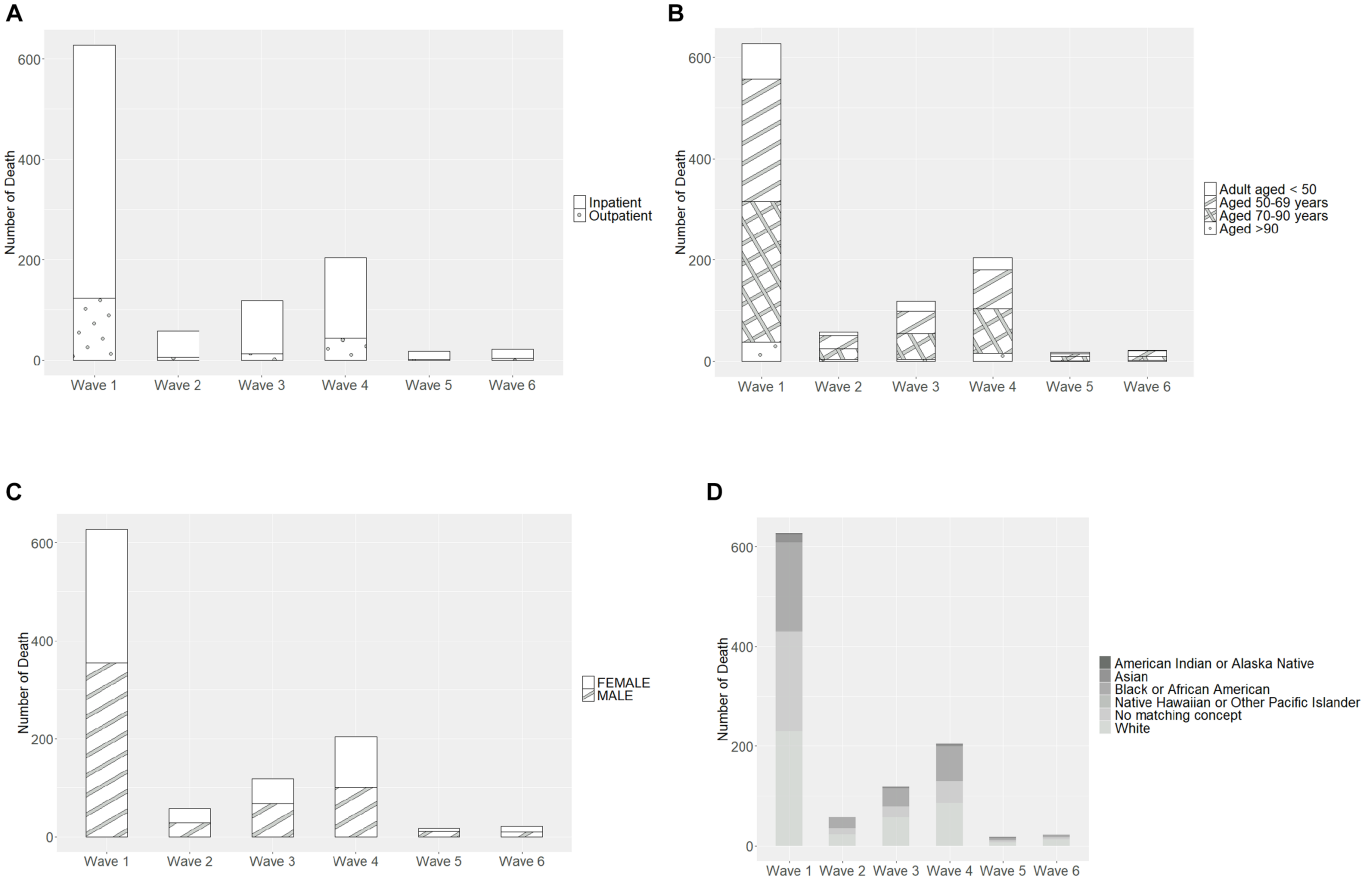
Characteristics of patients recorded as death in each pandemic wave, by **(A)** care setting, **(B)** age group, **(C)** sex, and **(D)** race. Wave 1: March 7, 2020 to March 20, 2021, Wildtype+D614G; Wave 2: March 21, 2021 to June 19, 2021, Alpha variant; Wave 3: June 20, 2021 to December 11, 2021, Delta variant; Wave 4: December 12, 2021 to March 19, 2022, Omicron BA.1 variant; Wave 5: March 20, 2022 to June 18, 2022, Omicron BA.2 variant; and Wave 6: June 19, 2022 to present (September 30, 2022), Omicron BA.4/BA.5 variant.

**Figure 3:**
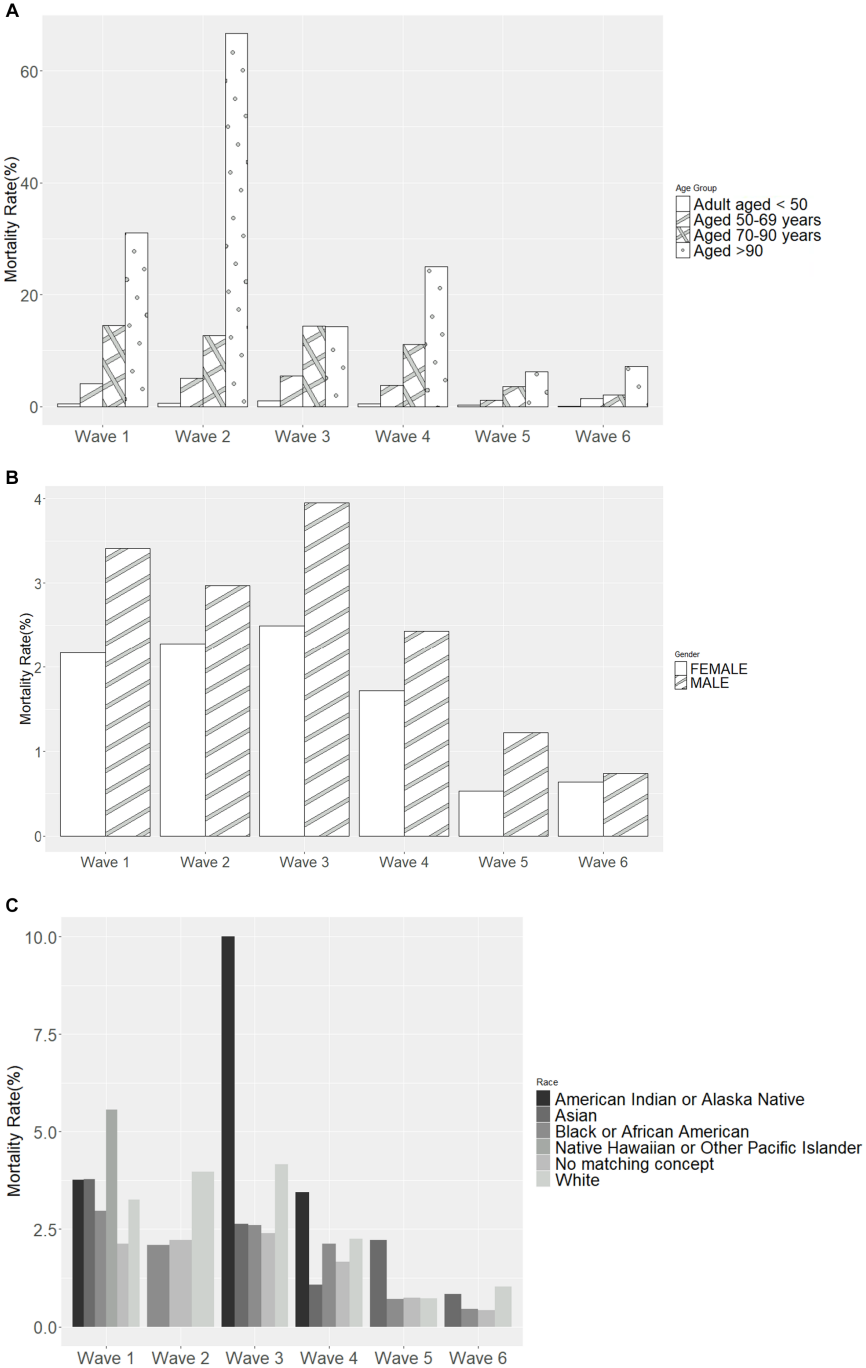
Mortality rate in each pandemic wave, by **(A)** age group, **(B)** sex, and **(C)** race. Wave 1: March 7, 2020 to March 20, 2021, Wildtype+D614G; Wave 2: March 21, 2021 to June 19, 2021, Alpha variant; Wave 3: June 20, 2021 to December 11, 2021, Delta variant; Wave 4: December 12, 2021 to March 19, 2022, Omicron BA.1 variant; Wave 5: March 20, 2022 to June 18, 2022, Omicron BA.2 variant; and Wave 6: June 19, 2022 to present (September 30, 2022), Omicron BA.4/BA.5 variant.

**Figure 4:**
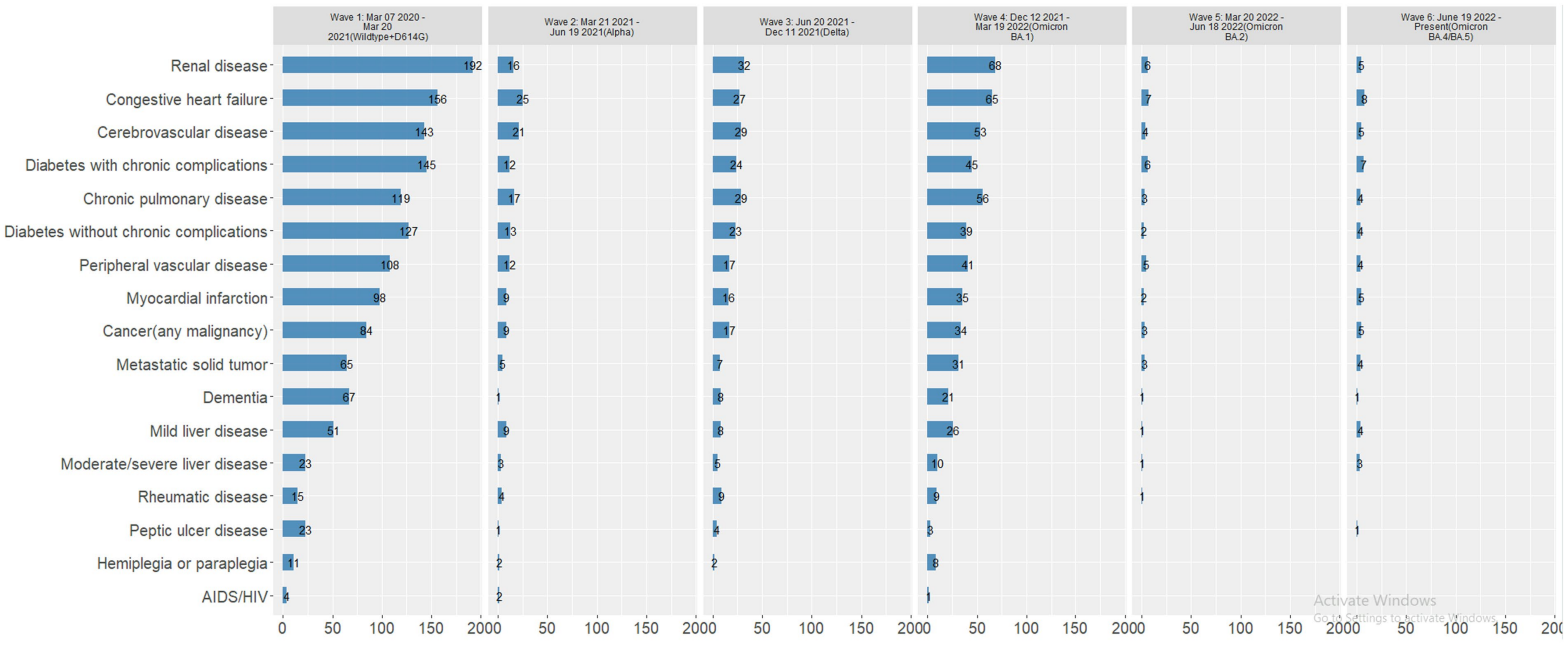
Number of patients reported as a death with comorbid conditions, by wave. Wave 1: March 7, 2020 to March 20, 2021, Wildtype+D614G; Wave 2: March 21, 2021 to June 19, 2021, Alpha variant; Wave 3: June 20, 2021 to December 11, 2021, Delta variant; Wave 4: December 12, 2021 to March 19, 2022, Omicron BA.1 variant; Wave 5: March 20, 2022 to June 18, 2022, Omicron BA.2 variant; and Wave 6: June 19, 2022 to present (September 30, 2022), Omicron BA.4/BA.5 variant.

Most deaths occurred in Wave 1 (*n* = 627, 59.9%), followed by Wave 4 (Omicron BA.1 variant; *n* = 204, 19.5%) (Table 4). The mortality rate ranged from 3.2% in Wave 3 to 0.8% in Wave 5 and 0.7% in Wave 6, with similar rates in Waves 1, 2, and 4 (2.7, 2.6, and 2.0%, respectively). In each wave, the majority of deaths occurred among inpatients (Figure 2A) and those 50–69 and 70–90 years of age (Figure 2B). The mortality rate was consistently highest among those 70–90 years of age in each wave (Figure 3A). Deaths were slightly more frequent in males than females in Waves 1, 3, and 5 (Figure 2C), with a consistently higher mortality rate among males in each of the 6 waves (Figure 3B). The proportion of deaths in Waves 1 and 2 were similar for White and Black or African American patients; in Waves 3–6, White patients represented the highest proportion of deaths (Figure 2D). Mortality rates were slightly higher for White patients compared to other racial groups in most waves, with the exception of Wave 1, where the mortality rate was highest among Native Hawaiian or Other Pacific Islanders and Waves 3 and 4, where the mortality rate was highest among American Indians or Alaska Natives (Figure 3C). Note however, that these 2 groups made up a very small proportion of patients in the registry. In every wave of the pandemic, the most common comorbid conditions among patients reported as a death were renal disease, congestive heart failure, cerebrovascular diseases, diabetes, and chronic pulmonary disease, with similar frequencies in each wave (Figure 4).

### 3.4 Age-stratified analyses for deaths across all waves

Because the mortality rate was higher among older patients and those with certain comorbidities, we examined the association between age and comorbid conditions in all COVID-19 positive patients in the registry and those reported as a death.

#### 3.4.1 Comorbidities by age

**Figure 5:**
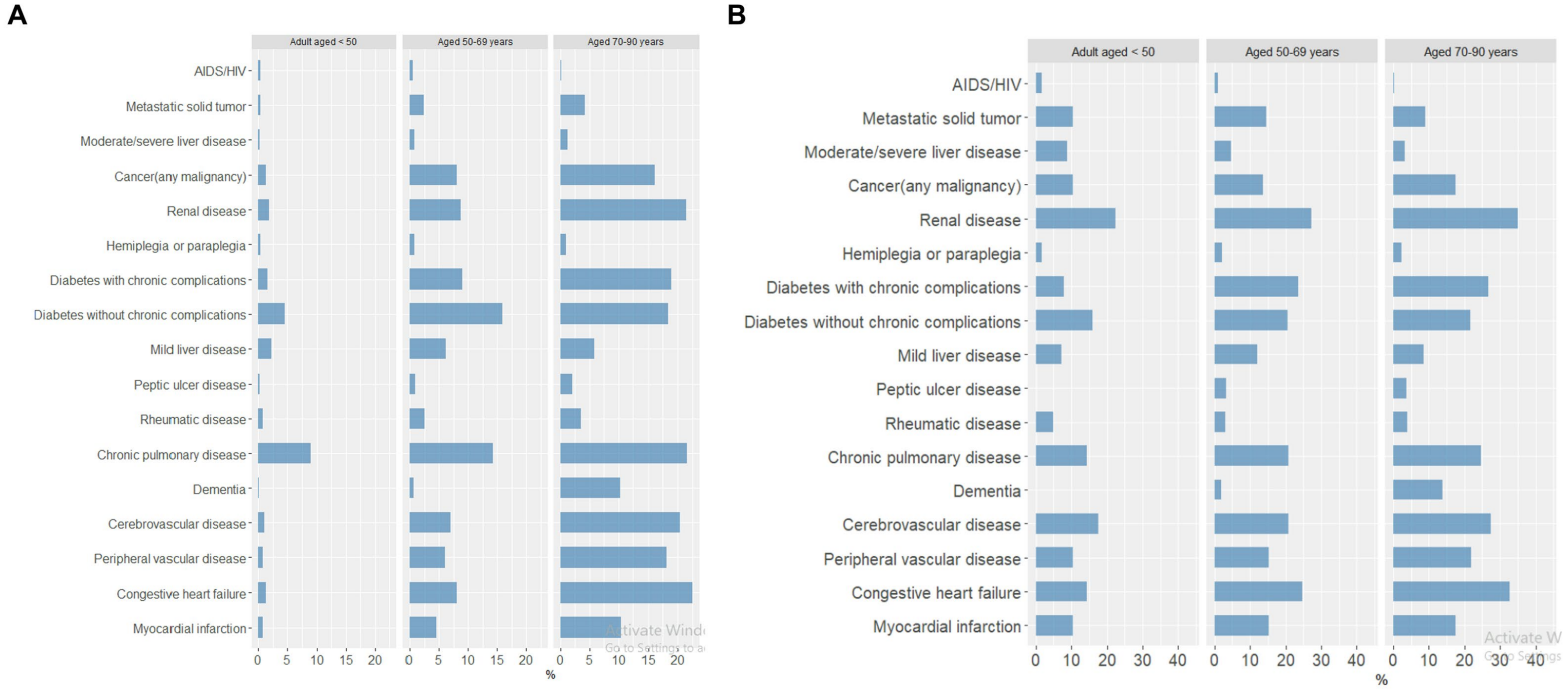
Percentage of patients with comorbid conditions by age group across all pandemic waves, in **(A)** COVID-19–positive patients (18–49 years, *n* = 23,990; 50–69 years, *n* = 10,540; 70–90 years, *n* = 3,860) and **(B)** patients reported as a death (18–49 years, *n* = 125; 50–69 years, *n* = 404; 70–90 years, *n* = 453).

The frequency of comorbid conditions increased with increasing age (Figure 5A). Chronic pulmonary disease was the most common comorbidity in all three age groups across all waves of the pandemic. Chronic pulmonary disease was the most common comorbidity in patients 18–49 years of age, while diabetes without chronic complications was the most common comorbidity for those 50–69 years of age, and congestive heart failure was the most common comorbidity for patients 70–90 years of age.

#### 3.4.2 Mortality for comorbidities by age

Among those patients reported as a death, the frequency of comorbid conditions was similar across age groups, with renal disease as the most common comorbidity in all three age groups (Figure 5B). Cerebrovascular disease was the second most common comorbidity for patients 18–49 years of age, whereas congestive heart failure was the second most common comorbidity for patients 50–69 and 70–90 years of age.

### 3.5 Age-stratified analyses in each wave

#### 3.5.1 Comorbidities by age and by wave

**Figure 6:**
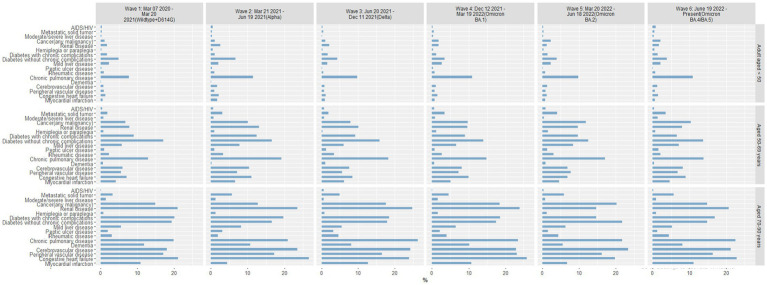
Percentage of COVID-19 positive patients with comorbid conditions by age group in each pandemic wave. Wave 1: March 7, 2020 to March 20, 2021, Wildtype+D614G; Wave 2: March 21, 2021 to June 19, 2021, Alpha variant; Wave 3: June 20, 2021 to December 11, 2021, Delta variant; Wave 4: December 12, 2021 to March 19, 2022, Omicron BA.1 variant; Wave 5: March 20, 2022 to June 18, 2022, Omicron BA.2 variant; and Wave 6: June 19, 2022 to present (September 30, 2022), Omicron BA.4/BA.5 variant.

In each wave of the pandemic, chronic pulmonary disease was consistently the most common comorbid condition in COVID-19–positive patients 18–49 years of age, and diabetes without chronic complications was the second most common comorbidity (Figure 6). In Wave 1, patients 50–69 years of age were most likely to have diabetes without chronic complications or chronic pulmonary disease; in subsequent waves, several other comorbid conditions were seen with increased prevalence, notably diabetes with chronic complications, renal disease, and any cancer. Patients in the oldest group (70–90 years) had several comorbidities with similarly high frequencies in each wave; congestive heart failure, chronic pulmonary disease, renal disease, and cerebrovascular disease were consistently high in each wave.

#### 3.5.2 Mortality for comorbidities by age and by wave

**Figure 7:**
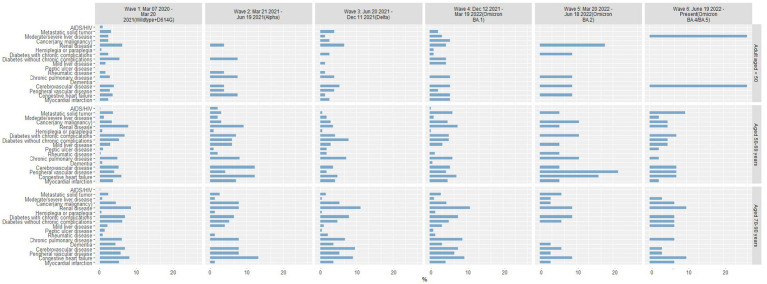
Percentage of patients reported as a death with comorbid conditions by age group in each pandemic wave. Wave 1: March 7, 2020 to March 20, 2021, Wildtype+D614G; Wave 2: March 21, 2021 to June 19, 2021, Alpha variant; Wave 3: June 20, 2021 to December 11, 2021, Delta variant; Wave 4: December 12, 2021 to March 19, 2022, Omicron BA.1 variant; Wave 5: March 20, 2022 to June 18, 2022, Omicron BA.2 variant; and Wave 6: June 19, 2022 to present (September 30, 2022), Omicron BA.4/BA.5 variant.

Among patients reported as a death, age-related comorbid conditions were slightly more frequent in the oldest age group in Waves 1–4, with consistent patterns of comorbid conditions among patients 50–69 and 70–90 years of age (Figure 7). Comorbid conditions within the youngest age group (18–49 years) varied widely with each pandemic wave. By Waves 5 and 6, the most common comorbid conditions were significantly different in each age group. In Wave 5, the youngest group of patients reported as a death were most likely to have renal disease, followed by diabetes with chronic complications, chronic pulmonary disease, cerebrovascular disease, and congestive heart failure. In contrast, patients 50–69 years of age were most likely to have peripheral vascular disease or congestive heart failure in Wave 5, whereas patients 70–90 years of age were most likely to have renal disease, diabetes with chronic complications, and congestive heart failure. Patterns of comorbid conditions in Wave 6 were similar for patients reported as a death in the 2 older age groups, though more patients 50–69 years of age presented with metastatic solid tumors and those 70–90 years of age most often presented with renal disease and congestive heart failure.

### 3.6 Multivariable logistic regression models

**Figure 8:**
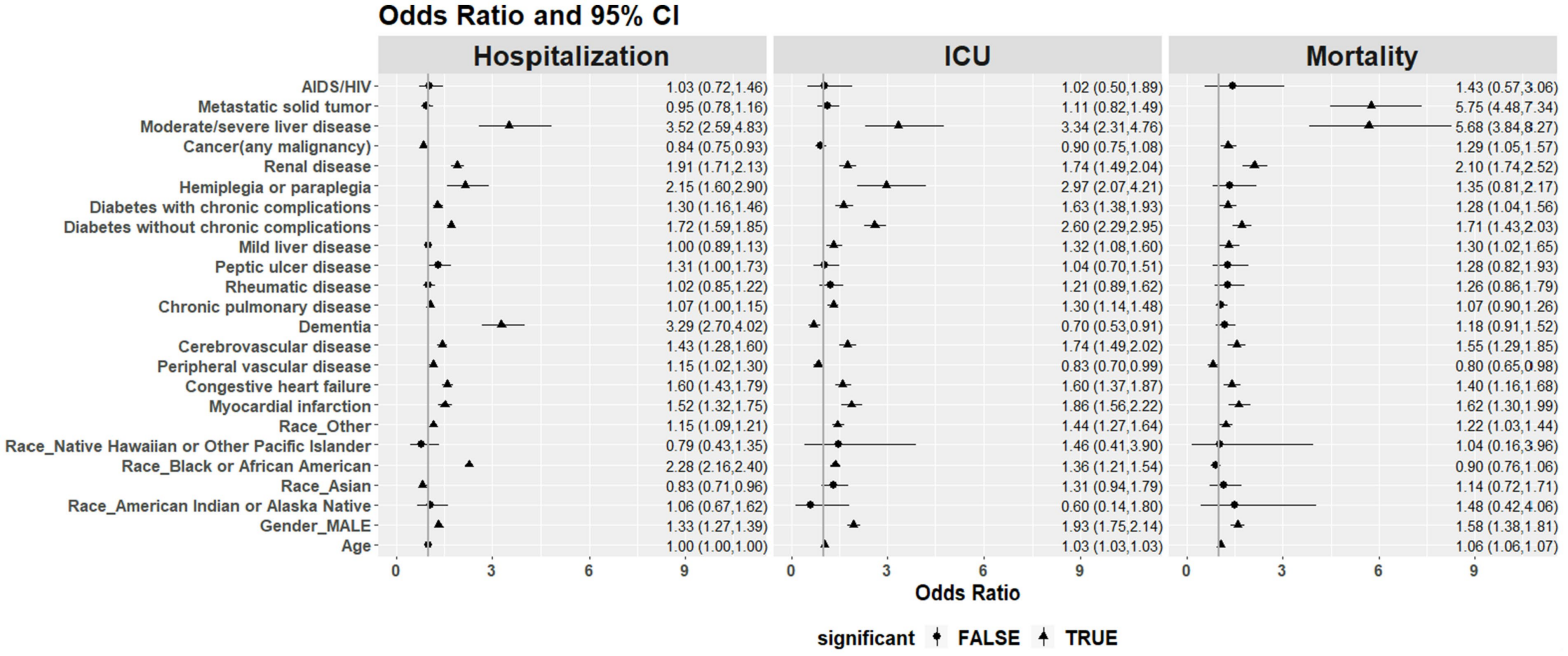
Odds ratio (OR) and 95% CI of the association of key factors with hospitalizations, ICU admissions, and mortality with each of the outcomes adjusted for comorbidities, sex, ethnicity, and age.

Multivariable logistic regression models were used to estimate the association of key factors with each of the outcomes adjusted for comorbidities, sex, ethnicity, and age (Figure 8). Liver disease was significantly associated with all outcomes: increased hospitalization (OR = 3.52, 95% CI 2.59–4.83), ICU admission (OR = 3.34, 95% CI 2.31–4.76), and mortality (OR = 5.68, 95% CI 3.84–8.27). Patients with metastatic solid tumors were more likely to be reported as a death (OR = 5.75, 95% CI 4.48–7.34). Dementia, being Black or African American, hemiplegia or paraplegia, and renal disease were found to be significantly associated with hospitalizations. Hemiplegia or paraplegia, renal disease, and diabetes with and without chronic complications were significantly associated with ICU admissions. Renal disease, myocardial infarction, and diabetes with and without chronic complications were significantly associated with mortality.

## 4 Discussion

This study leveraged the rich dataset from the RUMC COVID-19 registry to analyze the association between age-related comorbid conditions and death across and within each wave of the COVID-19 pandemic since March 2020. While several studies have examined risk factors associated with hospitalizations, ICU admissions, and death, few have evaluated differences in these factors across distinct waves of the pandemic (18–22). The Alpha and Delta variants were associated with greater hospitalizations, ICU admissions, and mortality compared to the other variants.

### 4.1 Overall mortality rates

Our finding of higher mortality rates in older patients, and a decrease in mortality across all age groups in later waves, is consistent with national trends (1). The association of mortality with renal disease, congestive heart failure, cerebrovascular disease, diabetes, and chronic pulmonary disease is also consistent with various reports in the US and elsewhere (23–26). These findings underscore the value of COVID-19 registries. Several studies have illustrated the advantage of adding COVID-19 data points to existing registries in specific populations—such as cancer (27), inflammatory diseases (28), neurological disorders, transplantation (29), and cardiovascular disease (30)—in order to examine predictors of risk for COVID-19 outcomes. Further, evaluation of COVID-19 and comorbidities has been reviewed by several authors and have found significant associations. Hospitalizations and mortality for other respiratory infections were significantly decreased during the pandemic (31). The effect of COVID-19 on STEMI (32) and stoke (33) outcomes have also been demonstrated, resulting in higher in-patient mortality, length of stay and cost of hospitalization. Kapuria et al. found that patients with COVID-19 and cirrhosis had 3 times higher mortality than those without COVID-19 (34) and Pal et al. found sex and racial disparities in hospitalizations and in rates of adverse outcomes (35).

### 4.2 Mortality rates by wave

In our age-stratified analyses of patients recorded as a death, we found notable differences in the prevalence of specific comorbid conditions in each age group that changed with each wave of the COVID-19 pandemic. For example, younger patients reported as a death presented with various comorbid conditions in early waves of the pandemic but were most likely to have renal disease in later waves. Older patients recorded as a death also presented with various comorbid conditions in consistent patterns across early waves of the pandemic, but by Wave 5, those 50–69 years of age more often presented with peripheral vascular disease and congestive heart failure and those 70–90 years of age presented with renal disease, diabetes, and congestive heart failure. The mechanisms driving these differences in age-related comorbid conditions in patients reported as a death is unknown but may be related to differences in the predominant variant, previous infection history, vaccination status, or other patient-related factors, and may be important factors to consider when developing interventions (25).

### 4.3 Limitations

Although the registry contained pediatric patients, the modeling of risk factors and mortality focused on an adult population. Several limitations should be considered and there are several bias sources to consider when using EHR data, particularly related to how data is collected as well as environmental aspects which can influence the quality of data (36). First, the retrospective design of the study has the potential for missing data and reporting bias, especially toward more severe symptoms or outcomes being reported more frequently. Comorbidities were potentially underreported in the registry, and one-third of patients reported an undisclosed racial group in the registry, which limited our ability to draw strong conclusions about race as a risk factor for mortality across and within individual pandemic waves. The registry did not collect information about vaccination status or measure changes to clinical practice in treating COVID-19 as the pandemic unfolded, although the analysis by wave is a surrogate measure for both important factors. The registry source lacked sequencing data to confirm variants; instead, the predominant variant circulating in Chicago and epidemiological assumptions was used to define waves. There also exists the potential that patients attended other healthcare institutions in the Chicagoland area, thus underreporting death that occurred outside of RUMC. Data in the EHR are susceptible to data coding errors and the subsequent mapping to OMOP common data model could compound this. Comorbid conditions were classified based on any history of the disease, but it could not be ascertained from the data whether the patients were still suffering from these conditions. Given that most of these comorbid conditions are chronic and lifelong, this is a potential but small bias. Information regarding co-infections with other respiratory viruses was also not known. Lastly, no single institution can be generalizable to the general patient population.

## 5 Conclusion

The findings of this specific COVID-19 registry within a single localized population were representative of the trends seen in the US; namely, higher mortality rates in older patients and a decrease in mortality across all age groups with later variant waves. The association of mortality with the most at risk populations due to specific comorbidities of renal disease, congestive heart failure, cerebrovascular disease, diabetes, and chronic pulmonary disease was also generalizable to the US.

Future research should evaluate and include the impact of vaccination status and co-infection with other circulating respiratory viruses, as well as the effect of long-COVID on hospitalization, ICU admittance and death.

Overall, the RUMC COVID-19 registry provided a valuable resource for population-based analyses to identify risk factors for death and other outcomes and to understand the evolution with each wave of the pandemic. Our findings may have implications for risk stratification and care planning for patients with COVID-19 based on age and presenting comorbid conditions for subsequent variant waves.

## Data availability statement

The data analyzed in this study is subject to the following licenses/restrictions: data is from Rush University Medical Center. Requests to access these datasets should be directed to Rush-affiliated authors.

## Ethics statement

The studies involving humans were approved by Rush University Medical Center Institutional Review Board. The studies were conducted in accordance with the local legislation and institutional requirements. Written informed consent for participation was not required from the participants or the participants’ legal guardians/next of kin because retrospective analysis of data. Written informed consent was not obtained from the individual(s), nor the minor(s)’ legal guardian/next of kin, for the publication of any potentially identifiable images or data included in this article because retrospective analysis of data.

## Author contributions

Y-sC: Formal analysis, Methodology, Validation, Visualization, Writing – review & editing. SG: Methodology, Project administration, Supervision, Writing – original draft, Writing – review & editing. PD: Methodology, Project administration, Supervision, Writing – original draft, Writing – review & editing. JR: Methodology, Supervision, Writing – review & editing. HB: Methodology, Writing – review & editing. NT: Data curation, Formal analysis, Methodology, Writing – review & editing. RG: Project administration, Resources, Supervision, Writing – review & editing. SS: Methodology, Project administration, Writing – review & editing. AO: Funding acquisition, Project administration, Resources, Supervision, Writing – original draft. GC: Funding acquisition, Resources, Supervision, Writing – review & editing. AL: Conceptualization, Supervision, Writing – review & editing.

## Footnotes

^1^ https://ohdsi.github.io/CommonDataModel/cdm531.html
